# Adaptive stimulus optimization for sensory systems neuroscience

**DOI:** 10.3389/fncir.2013.00101

**Published:** 2013-06-06

**Authors:** Christopher DiMattina, Kechen Zhang

**Affiliations:** ^1^Program in Psychology, Florida Gulf Coast UniversityFort Myers, FL, USA; ^2^Department of Biomedical Engineering, The Johns Hopkins University School of MedicineBaltimore, MD, USA

**Keywords:** sensory coding, optimal stimulus, adaptive data collection, neural network, parameter estimation

## Abstract

In this paper, we review several lines of recent work aimed at developing practical methods for adaptive on-line stimulus generation for sensory neurophysiology. We consider various experimental paradigms where on-line stimulus optimization is utilized, including the classical *optimal stimulus* paradigm where the goal of experiments is to identify a stimulus which maximizes neural responses, the *iso-response* paradigm which finds sets of stimuli giving rise to constant responses, and the *system identification* paradigm where the experimental goal is to estimate and possibly compare sensory processing models. We discuss various theoretical and practical aspects of adaptive firing rate optimization, including optimization with stimulus space constraints, firing rate adaptation, and possible network constraints on the optimal stimulus. We consider the problem of system identification, and show how accurate estimation of non-linear models can be highly dependent on the stimulus set used to probe the network. We suggest that optimizing stimuli for accurate model estimation may make it possible to successfully identify non-linear models which are otherwise intractable, and summarize several recent studies of this type. Finally, we present a two-stage stimulus design procedure which combines the dual goals of model estimation and model comparison and may be especially useful for system identification experiments where the appropriate model is unknown beforehand. We propose that fast, on-line stimulus optimization enabled by increasing computer power can make it practical to move sensory neuroscience away from a descriptive paradigm and toward a new paradigm of real-time model estimation and comparison.

## INTRODUCTION

One classical approach in sensory neurophysiology has been to describe sensory neurons in terms of the stimuli that are most effective to drive these neurons. The stimulus that elicits the highest response is often referred to as the *optimal stimulus* (Albrecht et al., 1980; Stork et al., 1982; DiMattina and Zhang, 2008). Although the optimal stimulus provides a simple and intuitive means of characterizing a sensory neuron, positively identifying the optimal stimulus may be technically difficult for high-dimensional stimuli, and simply knowing the optimal stimulus without adequately exploring responses to other stimuli may provide limited information about sensory function (Olshausen and Field, 2005). Due to these practical and conceptual limitations of characterizing neurons by the optimal stimulus, many researchers have recently taken engineering-inspired approaches to studying neural coding, for example, by characterizing neurons in terms of the mutual information between sensory stimuli and a neuron’s responses (Machens, 2002; Sharpee et al., 2004; Machens et al., 2005; Chase and Young, 2008), by characterizing iso-response surfaces in stimulus parameter spaces (Bölinger and Gollisch, 2012; Horwitz and Hass, 2012), or by fitting predictive mathematical models of neural responses to neurophysiology data (Wu et al., 2006). However, just like the classical optimal stimulus paradigm, these engineering-inspired methods also give rise to non-trivial high-dimensional stimulus optimization problems.

With recent advances in desktop computing power, it has become practical to perform stimulus optimization adaptively in real-time during the course of an experiment (Benda et al., 2007; Newman et al., 2013). In this review, we consider several recent lines of work on adaptive on-line stimulus optimization, focusing on single-unit recording *in vivo* for systems-level sensory neuroscience. Other kinds of closed-loop neuroscience experiments like dynamic patch clamping or closed-loop seizure interventions are considered elsewhere (Prinz et al., 2004; Newman et al., 2013). We first discuss the concept of the optimal stimulus and consider how its properties may be constrained by the underlying functional model describing a neuron’s stimulus–response relation. We then discuss how adaptive stimulus optimization has been utilized experimentally to find complex high-dimensional stimuli which optimize a neuron’s firing rate, including promising recent studies using evolutionary algorithms. We also discuss a different kind of study where stimuli are “optimized” to elicit a desired constant firing rate so that iso-response contours of the stimulus–response function may be obtained, as well as studies seeking maximally informative stimulus ensembles. Finally, we discuss how adaptive stimulus optimization can be utilized for effective estimation of the parameters of sensory processing models, as well as for effective model comparison. In conclusion, we suggest that adaptive stimulus optimization cannot only make the classical optimal stimulus paradigm more tractable, but can potentially move sensory neuroscience toward a fundamentally new experimental paradigm of real-time model estimation and comparison.

## THE OPTIMAL STIMULUS

### DEFINING THE OPTIMAL STIMULUS

In order for a sensory neuron to be useful to an organism, there must be a consistent functional relationship between the parameters of sensory stimuli and neural responses. Although this relationship may be highly complex and non-linear, for any set of stimuli defined by parameters **x** = (*x*_1_,....,*x*_*n*_)^T^ we may think abstractly of the expected neural responses being described by some function *r* = *f*(**x**). For simplicity and definiteness, in this section we will focus our discussion of the optimal stimulus on the most common case where *r* is a scalar quantity which represents the firing rate of a single neuron, and will assume that the expected firing rate is entirely a function of the stimulus parameters, ignoring variables such as spiking history and stimulus-specific adaptation by assuming that they are kept constant (Ulanovsky et al., 2003; Bartlett and Wang, 2005; Asari and Zador, 2009).

**FIGURE 1:**
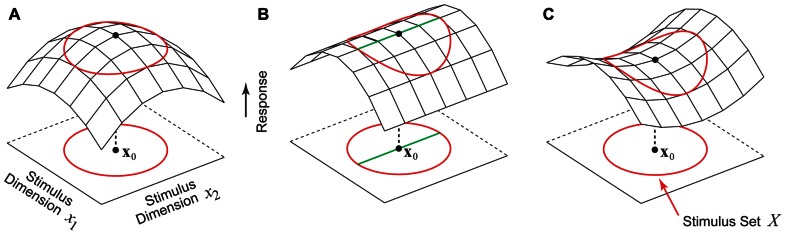
**Hypothetical stimulus–response relationships for a sensory neuron**. The red circle represents the boundary of the set of permissible stimuli. **(A)** Stimulus **x**_0_ is a firing rate peak which corresponds to the intuitive notion of the optimal stimulus where any perturbation away from **x**_0_ results in a decrease in the firing rate. **(B)** This neuron is tuned to one stimulus dimension but is insensitive to the second dimension. Instead of a single optimal stimulus **x**_0_ there is a continuum of optimal stimuli (green line). **(C)** A neuron whose stimulus–response function around the point **x**_0_ is saddle-shaped. Along one stimulus dimension **x**_0_ is a firing rate maximum, and along the other stimulus dimension **x**_0_ is a minimum.

Given this formulation, the problem of finding the optimal stimulus **x**_0_ is simply the problem of maximizing the function *f*(**x**). Perhaps the simplest and most intuitive notion of the optimal stimulus is that of a firing rate peak in stimulus parameter space centered at **x**_0_, as illustrated in **Figure 1A**. Here *f* is maximized at **x**_0_, and for any stimulus perturbation Δ**x** we have *f*(**x**_0_ + Δ**x**) < *f*(**x**_0_). However, for high-dimensional stimulus spaces like image pixel space (Simoncelli et al., 2004) or auditory frequency space (Yu and Young, 2000; Barbour and Wang, 2003a) this intuitive notion of the optimal stimulus as a response peak is hardly the only possibility. In the example shown in **Figure 1B**, the neuron is tuned along one direction in the stimulus space, but is untuned along an orthogonal direction. In this case, there is not a single optimal stimulus **x**_0_ as in **Figure 1A**, but rather a continuum of optimal stimuli lying along a ridge containing x_0_ (**Figure 1B**, thick green line). Another theoretical possibility is the saddle-shaped response surface in **Figure 1C**, where depending on the dimension chosen for exploration, the same stimulus x_0_ can be either a firing rate peak or a valley.

For high-dimensional stimulus spaces, a full factorial exploration is impossible since the number of stimuli needed grows exponentially with the dimension, a problem referred to colloquially as the *curse of dimensionality* (Bellman, 1961). In many experiments, stimulus spaces are explored in a restricted subset of dimensions. The behaviors of neuron in the unexplored stimulus dimensions may have various possibilities including the ones considered above. One cannot assume that the stimulus–response relationship must always be a single peak as in **Figure 1A**. Indeed, one of the challenges of sensory neurophysiology is that without prior knowledge about the neuron under study, there are no constraints whatsoever on the possibilities for the optimal stimulus, which must be found in a process of trial-and-error with no way to conclusively prove global optimality (Olshausen and Field, 2005). We now briefly discuss a recent theoretical study describing possible constraints on the optimal stimulus which arise from general anatomical properties of underlying functional circuitry.

### CONSTRAINTS FROM UNDERLYING FUNCTIONAL CIRCUITRY

Ultimately, the stimulus–response relationship function *f*(**x**) is generated by the underlying neural circuitry connecting the sensory periphery to the neuron under study, but in general this circuitry is highly complex (Felleman and Van Essen, 1991; Shepherd, 2003) and not generally known to the experimenter. Nevertheless, recent theoretical work suggests that very basic anatomical properties of the neural circuitry may be able to provide experimentally useful constraints on the possibilities for the optimal stimulus (DiMattina and Zhang, 2008).

**FIGURE 2:**
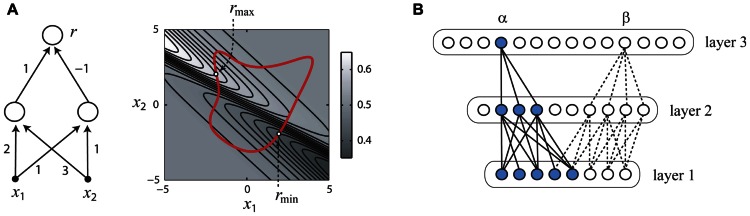
**How the optimal stimulus properties of sensory neurons may be constrained by network architecture**. Panels **(A,B)** adapted with permission from DiMattina and Zhang (2008). **(A)** A simple neural network (left) and its responses to inputs *x*_1_, *x*_2_ (right). The optimal stimulus for this network must lie on the boundary of any closed set of stimuli (right panel, thick red line). **(B)** The functional network connecting a single neuron (α or β) to the sensory periphery may have fewer units in successive processing layers (convergent), even if the overall number of neurons in successive processing layers is increasing (divergent).

Consider the simple hypothetical sensory network shown in **Figure 2A** (left panel) which receives synaptic inputs from two peripheral sensory receptors (filled black circles) which linearly transduce stimulus the variables *x*_1_, *x*_2_ and pass their outputs to a pair of interneurons, which in turn converge onto the output neuron from which responses *r* are measured. Since there are physical limits on the intensities of stimuli which can be generated by laboratory equipment, we may reasonably assume that the collection *X* of permissible stimuli is some closed subset of the real plane consisting of an interior and boundary (rightmost panel, thick red line). We may also reasonably assume that each neuron’s input–output property is a described by an increasing gain function *g*(*u*). With these reasonable assumptions, it is simple to show that that the gradient of the function *f*(**x**) implemented by this circuit cannot vanish, and thus an optimal stimulus which is a firing rate peak as in **Figure 1A** is impossible. Therefore, it follows that optimal stimulus must lie on the boundary of *X* (**Figure 2A**, right panel), with the exact location depending on the synaptic weights and other parameters of the network.

In general, it can be shown that for hierarchical neural networks which can be arranged into layers that if the gain functions are increasing, the number of neurons in successive layers is decreasing or constant, and weight matrices connecting successive layers are non-degenerate, then it is impossible for the optimal stimulus for any neuron in this network to be a firing rate peak like that illustrated in **Figure 1A** (DiMattina and Zhang, 2008). It is important to note that this result requires that the stimuli be defined in the space of activities of the input units to the neural network, such as image pixel luminances which are the inputs to the network. One interesting corollary of this result is that if the space *X* of permissible stimuli is bounded by a maximum power constraint $\Sigma_{i=1}^{n}x_{i}^{2}\leq E$, the optimum firing rate will be obtained for a stimulus **x** ∈ *X* having the greatest power or contrast, since this stimulus will lie on the boundary. Indeed, for many sensory neurons in the visual, auditory, and somatosensory modalities, increasing the stimulus contrast monotonically increases the firing rate response (Albrecht and Hamilton, 1982; Cheng et al., 1994; Oram et al., 2002; Barbour and Wang, 2003b; Ray et al., 2008), which is interesting considering that convergent networks satisfying the conditions of the theorem can model the functional properties of many sensory neurons (Riesenhuber and Poggio, 1999, 2000; Lau et al., 2002; Prenger et al., 2004; Cadieu et al., 2007).

At first, this result may seem to be of limited applicability since it is well known that the numbers of neurons in successive processing stages can be widely divergent (Felleman and Van Essen, 1991). However, the theorem applies only to the *functional network* which connects a given neuron to the sensory periphery. For instance, in the example shown in **Figure 2B**, the functional network connecting neuron *a *to the input layer is a convergent network with the number of units decreasing from layer to layer (blue), whereas the full network is divergent with the number of units increasing from layer to layer. Similarly, it is important to note that the neural network to which we apply the theorem may not be a literal description of the actual neural circuit, but simply a mathematical description of the functional relationship between the stimulus parameters and the neural response. For instance, a standard functional model of the ventral visual stream postulates a feedforward architecture similar to the models of complex cells postulated by Hubel and Wiesel (Riesenhuber and Poggio, 1999, 2000), and the theorem can be applied to neurons in these models. Similarly, divisive normalization models postulated for visual and auditory neurons (Heeger, 1992b; Koelling and Nykamp, 2012) can be re-written in a form to which the theorem applies and shown to have a non-vanishing gradient (Koelling and Nykamp, 2012).

## ADAPTIVE OPTIMIZATION OF FIRING RATE

Despite the conceptual difficulties with the notion of an optimal stimulus, it provides sensory neuroscience with an intuitive first-pass description of neural function when an appropriate quantitative model is unknown. In this section, we discuss adaptive stimulus optimization methods which have been utilized experimentally for optimizing the firing rate of sensory neurons in high-dimensional stimulus spaces where a full factorial exploration would be intractable. Mathematically, the optimization problem may be specified as that of finding

(1) $$\begin{array}{c} x^{*}=\underset{x\in X}{\arg\max}f\left(x\right), \end{array}$$

where x* is the optimal stimulus, *f* is the (unknown) stimulus–response function, and *X* is the set of allowable stimuli. Methods to optimize firing rate fall into two general categories: those that ascend the local gradient of the stimulus–response function, and those which utilize genetic or evolutionary approaches. We discuss each of these approaches and their relative merits, along with issues of adaptation and constrained stimulus spaces.

### LOCAL HILL-CLIMBING

Due to the inherent variability in neural responses (Tolhurst et al., 1983; Rieke et al., 1997), optimizing the firing rate of sensory neurons is a difficult stochastic optimization problem (Spall, 2003). Early work on adaptive stimulus optimization was performed by Harth and Tzanakou (1974), who applied a method of stochastic gradient ascent known as ALOPEX, or “Algorithm of Pattern Extraction” to neurons in the frog visual tectum (Tzanakou et al., 1979). This method works by computing correlations between random perturbations of the current stimulus and changes in firing rate and using these correlations to iteratively update the current stimulus to increase the expected firing rate, eventually converging to the optimal stimulus. More recently, related methods have been employed to optimize the responses of neurons in the primary visual (Foldiak, 2001) and auditory (O’Connor et al., 2005) cortices, providing independent verification of previously described receptive field properties like orientation selectivity (Hubel and Wiesel, 1962) or inhibitory sidebands (Shamma et al., 1993). Variations of ALOPEX have also been utilized to quickly find the best frequency for auditory nerve fibers, an essential first step in many auditory neurophysiology experiments (Anderson and Micheli-Tzanakou, 2002).

In addition to these correlation-based approaches, numerous other computational methods have been utilized for firing rate optimization. One approach is to iteratively make local linear or quadratic approximations to the neural responses around a reference stimulus (Bandyopadhyay et al., 2007a; Koelling and Nykamp, 2008, 2012), which can then be used to determine a good search directions in the stimulus space. This approach has been utilized by Young and colleagues in order to determine that the optimal stimulus for neurons in the dorsal cochlear nucleus is a spectral edge centered at the neuron’s best frequency (Bandyopadhyay et al., 2007b), consistent with suggestions from previous studies (Reiss and Young, 2005). An alternative optimization method which does not require estimating the local response function gradient is the Nelder–Mead simplex search (Nelder and Mead, 1965), which has been used to optimize the responses of neurons in cat auditory cortex to four-tone complexes (Nelken et al., 1994).

### GENETIC ALGORITHMS

One limitation of the stimulus optimization methods above is that they are local searches which iteratively update the location of a single point (or simplex of points). Therefore, it is certainly possible for optimization runs to end up stuck at local firing rate maxima. Furthermore points of vanishing gradient do not necessarily indicate maxima (Koelling and Nykamp, 2012), as we can see from the examples in **Figure 1**. Furthermore, local search methods only identify a single optimal stimulus, and do not sample the stimulus space richly enough to fully describe neural coding. One possible alternative adaptive optimization method used in previous neurophysiological studies which can potentially surmount both of these problems is a *genetic algorithm* (Goldberg, 1989). A genetic algorithm works by populating the stimulus space widely with many stimuli (analogous to “organisms”), which survive to the next generation with a probability proportional to the firing rate they elicit (analogous to their “fitness”). The parameters of the surviving stimuli are combined at random in a factorial manner (“crossing-over”) and mutated in order to produce a new generation of different stimuli based on the properties of the current generation. Over several iterations of this algorithm, a lineage of stimuli will evolve which maximizes the firing rate of the neuron under study, and since the sampling of the stimulus space is non-local, genetic algorithms are more likely to avoid the problem of local maxima than hill-climbing methods.

Genetic algorithms were applied to neurophysiology studies by Winter and colleagues, who optimized the parameters of amplitude-modulated tones defined in a four-dimensional space in order to study neural coding in the inferior colliculus (Bleeck et al., 2003). The optimal stimuli found by this method were in agreement with tuning functions found by traditional methods, thereby validating the procedure. More recently, a very powerful demonstration of genetic algorithms as a tool for adaptive optimization was given by Connor and colleagues studying the representation of two-dimensional shape in V4 (Carlson et al., 2011) and three-dimensional shape in the inferotemporal cortex (Yamane et al., 2008; Hung et al., 2012). The parameter space needed to define three-dimensional shapes is immense and impossible to explore factorially, with most of the stimuli in this space being ineffective. Nevertheless, a genetic algorithm was successful at finding shape stimuli having features which were effective at driving neurons, with the optimization results being consistent over multiple runs. Furthermore, because the genetic algorithm cross-over step generates stimuli which factorially combine different stimulus dimensions, it did a sufficiently thorough job of sampling the stimulus space to permit the investigators to fit predictive models which accurately described the tuning of the neurons to arbitrary shape stimuli (Yamane et al., 2008).

As reviewed above, the different methods developed for automatically optimizing firing rate responses of sensory neurons differ greatly, both in their general search strategy (i.e., gradient ascent versus genetic algorithms) as well as their exact methods for implementing that strategy (Nelken et al., 1994; Foldiak, 2001; Koelling and Nykamp, 2012). Furthermore, it is important to note that while genetic algorithms are a commonly chosen alternative to gradient ascent in the existing literature (Bleeck et al., 2003; Yamane et al., 2008; Chambers et al., 2012; Hung et al., 2012), a wide variety of alternative optimization methods could in principle be applied as well, such as simulated annealing (Kirkpatrick et al., 1983), and particle swarm optimization (Kennedy and Eberhart, 1995). However, without direct comparisons of algorithms on benchmark problems using numerical simulation, it is hard to directly and fairly compare these various methods. As automated stimulus optimization becomes more widely used in physiological experiments, systematic comparison of optimization methods on benchmark problems is certainly an interesting avenue for future research in computational neuroscience.

### STIMULUS SPACE CONSTRAINTS

Quite often, one may wish to optimize neuronal responses in a constrained stimulus space for constraints which are more complex than simple upper and lower bounds on stimulus dimensions. For many neurons one can always increase the firing rate simply by increasing the stimulus energy or contrast (Albrecht and Hamilton, 1982; Cheng et al., 1994; Oram et al., 2002), so it is of interest to optimize the stimulus with the constraint of fixed stimulus energy. In Eq. 1, the optimal stimulus is defined over the set of all allow- able stimuli, *X*, which depends on the constraints in the stimulus space. When each component of the stimulus **x** = (*x*_1_,…, *x_n_*)^T^ is constrained between an upper bound and a lower bound (e.g., the luminance of image pixels has a limited range of possible values), the set *X* is a hypercube:

(2) $$\begin{array}{c} X=\left\{x:a_{i}\leq x_{i}\leq b_{i},i=1,...,n\right\}. \end{array}$$

With a quadratic energy constraint, the allowable stimulus set *X* should become a hyper-sphere:

(3) $$\begin{array}{c} X=\left\{x:\overset{n}{\underset{i=1}{\Sigma }}x_{i}^{2}=E\right\}. \end{array}$$

For example, Lewi et al. (2009) derived a fast procedure for optimization for effective model estimation under stimulus power constraints. Optimizing the stimulus in Eq. 1 subject to an energy constraint is an optimization problem for which there are many numerical methods for solutions (Douglas et al., 2000; Nocedal and Wright, 2006).

In special cases where there is prior information about the functional form of *f*(**x**), the constrained optimization problem may permit numerically elegant solutions for finding optimal stimuli subject to non-linear constraints, as well as finding invariant transformations of a stimulus which leave responses unchanged. A recent study (Berkes and Wiskott, 2006, 2007) considered the problem of optimizing the responses of any neuron whose functional properties are given by an inhomogeneous quadratic form f(**x**) = **x**^T^**Ax** + **b**^T^**x** + *c*, subject to an energy constraint **x**^T^**x** = *E*. This study presented a very efficient algorithm for computing the optimal stimulus x* which requires only a bounded one-dimensional search for a Lagrange multiplier, followed by analytical calculation of the optimal stimulus. In addition, they demonstrated a procedure for finding approximate invariant transformations in the constrained stimulus space, which for complex cells amount to shifts in the phase of a Gabor patch. As quadratic models have become popular tools for characterizing non-linear sensory neurons (Heeger, 1992a; Yu and Young, 2000; Simoncelli et al., 2004; Berkes and Wiskott, 2005; Bandyopadhyay et al., 2007a), their algorithm offers a useful tool for sensory neuroscience.

### NEURAL RESPONSE ADAPTATION

It is well known that when the same stimulus is presented repeatedly to sensory neurons, they exhibit firing rate adaptation, becoming less sensitive to that stimulus over time (Ulanovsky et al., 2003; Asari and Zador, 2009). Similarly, responses to sensory stimuli can often non-stationary and are affected by context provided by preceding stimuli (Bartlett and Wang, 2005). Adaptation potentially presents a difficulty for stimulus optimization methods, since toward the end of the optimization run as the algorithm converges on a (locally) optimal stimulus, a series of very similar stimuli may be presented repeatedly, thereby leading to firing rate adaptation. This phenomena has been observed in studies in the published literature (Yamane et al., 2008) and presents a potential obstacle to studies of adaptive stimulus optimization (Koelling and Nykamp, 2012). Given the suppression of neural responses to stimuli which occur with high probability (Ulanovsky et al., 2003), one way of dealing with adaptation may be to intersperse random stimuli with those generated by the optimization run, so as to reduce adaptation effects. However, this may be an inefficient method for dealing with adaptation, since it increases the number of stimuli needed in an experiment (Koelling and Nykamp, 2012).

Apart from these technical considerations, the problem of firing rate adaptation illustrates a fundamental conceptual limitation of phenomenological sensory neurophysiology. In particular, it demonstrates that the act of probing a sensory neuron with stimuli can potentially changes the response properties of the neuron itself, possibly including its optimal stimulus. Therefore, it may not be conceptually correct to characterize the stimulus optimization problem as it is written in Eq. 1, but rather to characterize it as a far more complicated optimization problem where the function *f*(**x**, **h** (*t*)) to be optimized is constantly changing, dependent on both the stimulus **x** and response history **h** (*t*). In this case, the optimal stimulus for a given neuron may only be well-defined with respect to a given history of stimuli and responses.

One solution to this problem would be to have a mathematical model of the neuron’s stimulus–response function which takes adaptation into account. Indeed, recent work has demonstrated that bilinear models of sensory neurons incorporating adaptation parameters can greatly improve predictions when compared standard linear receptive field models (Ahrens et al., 2008a). Other work has shown that the failure of spectrotemporal receptive field (STRF) models to account fully for neural responses to natural stimuli may be accounted for by rapid synaptic depression (David et al., 2009), further underscoring the importance of including adaptation parameters in neural models. We discuss the issues of neuronal adaptation and stimulus-response history further when we discuss the estimation of neural models using active data collection. 

On the whole however, the problem of adaptation does not seem to pose a fatal limitation to adapting firing rate optimization, as it has been applied successfully in many recent studies (Foldiak, 2001; O’Connor et al., 2005). Furthermore, there are many neurons in the brain for which adaptation effects are small and thus do not pose a concern (Ingham and McAlpine, 2004). These methods are potentially of great importance for investigating neural coding of complex stimuli defined in high-dimensional spaces (Yamane et al., 2008), and it is of great interest to better understand how adaptation affects stimulus optimization and receptive field characterization.

## ISO-RESPONSE SURFACES AND MODEL COMPARISON

**FIGURE 3:**
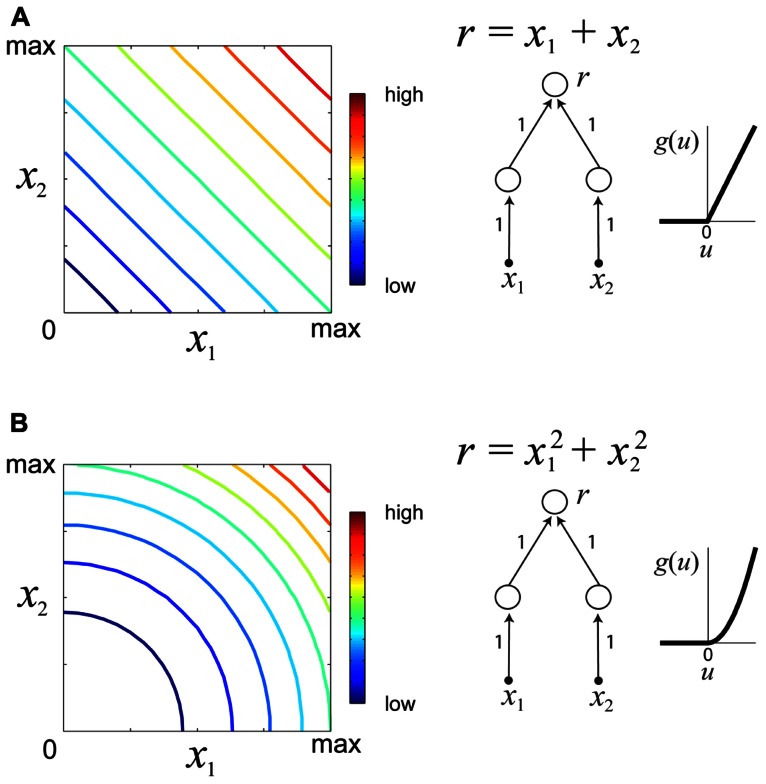
**Examples of iso-responses surfaces for two hypothetical sensory processing models**. **(A)** Iso-response contours (left) of a sensory neuron which linearly integrates stimulus variables *x*_1_, *x*_2_ ≥ 0. The response *r* of this neuron is a summation of the outputs of two neurons in the lower layer with a threshold-linear gain function (right). Colors in the contour plot represent neural firing rates from low to high. **(B)** Iso-responses contours (right) of a sensory neuron which non-linearly integrates stimulus variables *x*_1_, *x*_2_ ≥ 0 with a threshold-quadratic gain function (right).

In high-dimensional stimulus spaces, the same response from a sensory neuron can be elicited by a continuum of equally effective optimal stimuli rather than a single optimal stimulus (**Figure 1**). Therefore, in some experiments it may be of interest to find sets of equivalent stimuli known as *iso-response surfaces* which yield the same response. One possible way of formalizing an optimization problem for this class of experiments is to formulate it as finding stimuli

(4) $$\begin{array}{c} x^{*}=\underset{x\in X}{\arg\min}\,d\left(f(x),c\right), \end{array}$$

which *d*(·,·) is some metric measure (e.g., squared error) quantifying the discrepancy between the desired response *c* and the neuronal response *f*(**x**). Multiple optimization runs from different starting locations and for different values of the desired constant response *c* permit the experimenter to determine families of iso-rate surfaces for the neuron under study. The geometrical shapes of the iso-rate surfaces can help to determine how stimulus variables *x*_1_,…,*x*_*n*_ are integrated, and thus provide a useful tool for comparison of hypothetical models. For instance, linear integration of stimulus energy would yield iso-response surfaces which are hyperplanes of the form $\Sigma_{i=1}^{n}x_{i}=c$, whereas non-linear integration would yield non-planar iso-response surfaces. **Figure 3** illustrates iso-response surfaces for two different hypothetical sensory processing models.

The iso-response surface method was used by Gollisch et al. (2002) to test several competing hypotheses about how spectral energy is integrated in locust auditory receptors. The iso-response contours to combinations of two or three pure tone stimuli with fixed frequencies and variable amplitudes were of elliptical shape, consistent with an energy-integrator model of spectral integration. Further work extended the iso-response method to incorporate temporal integration, yielding a complete cascade model of auditory transduction (Gollisch and Herz, 2005).

A more recent study applied this technique to study the integration of visual contrast over space in salamander retinal ganglion cells, revealing a threshold-quadratic non-linearity in the receptive field center as well as a subset of ganglion cells most sensitive to spatially homogeneous stimuli (Bölinger and Gollisch, 2012). The iso-response surface method has also been applied fruitfully in mammalian sensory systems as well. A recent study by Horwitz and Hass (2012) utilized this procedure to study integration of color signals from the three retinal cone types in single neurons in the primary visual cortex. It was found that half of the neurons had planar iso-response surfaces, consistent with linear integration of cone signals. However, the other half showed a variety of non-linear iso-response surfaces, including cup-shaped surfaces indicating sensitivity to only narrow regions of color space.

Although the iso-response surface method has been applied successfully in stimulus spaces of low dimensionality (two or three dimensions), tracing out level hyper-surfaces in higher-dimensional spaces may pose a formidable computational challenge (Han et al., 2003; Willett and Nowak, 2007). In future research, dimensionality reduction procedures might be useful for extending the iso-response surface method to high-dimensional stimulus spaces like pixel space or auditory frequency space (Yu and Young, 2000), as well as for high-dimensional spaces defining complex naturalistic stimuli like 3D shapes or species-specific communication sounds (DiMattina and Wang, 2006; Yamane et al., 2008).

## MAXIMALLY INFORMATIVE STIMULUS ENSEMBLES

It has been proposed that one of the major goals of sensory coding is to efficiently represent the natural environment (Barlow, 1961; Simoncelli, 2003). In this spirit, another class of closed-loop stimulus optimization methods has been developed to find the optimal *ensemble* of sensory stimuli for maximizing the mutual information between stimuli and neural responses (Machens, 2002). This method differs from efforts to find the optimal stimulus or efforts to find iso-response surfaces because the goal is not to find an individual stimulus x* which optimizes the desired criterion (i.e., Eq. 1), but rather to find the optimal distribution p*(**x**) which optimizes the mutual information *I*(y; **x**), where y denotes the observed neural response (typically the firing rate of a single neuron). Mathematically, we write

(5) $$\begin{array}{c} p^{*}(x)=\underset{p(x)\in P}{\arg\max}\,I(y;x)=\int_{X}\int_{Y}p\left(y|x\right)p\left(x\right)\ln\frac{p\left(y|x\right)}{p\left(y\right)}dxdy, \end{array}$$

where *P* is the space of probability densities on the stimulus space *X*, and *p*(*y* | **x**) and *p*(*y*) are determined experimentally by observing neural responses to stimuli. In practice, one starts with an assumption of a uniform distribution with finite support and then applies the Blahut–Arimoto algorithm (Blahut, 1972; Arimoto, 1972) to iteratively update the sampling weights (Machens, 2002). This method has been applied experimentally to characterize grasshopper auditory receptor neurons, demonstrating optimality for processing behaviorally relevant species-specific communication sounds (Machens et al., 2005; Benda et al., 2007).

## ADAPTIVE OPTIMIZATION FOR SENSORY MODEL ESTIMATION

An ideal gold standard for sensory neuroscience is to obtain a complete and accurate functional stimulus–response model of the neuron under study. In theory, once such a model is attained, one can then numerically or analytically calculate from this model the neuron’s optimal stimulus, its iso-response surfaces, and its maximally informative stimulus ensembles. That is, if one identifies the system, one gets “for free” other information one may be interested in. However, despite its conceptual appeal, the problem of system identification is of great practical difficulty. This is because one needs to specify an accurate yet experimentally tractable model whose parameters can be estimated from data obtained during the available observation time. Unfortunately, research in computational neuroscience has shown that tractable (e.g., linear and quadratic) models are not accurate, whereas more biologically accurate models (deep, multiple layer neural networks incorporating dynamics, recurrence, etc.) often pose intractable parameter estimation problems.

It is well known from the fields of statistics and machine learning that one can more quickly and accurately estimate the parameters of a function using adaptive data collection, where new observations are generated in an iterative, adaptive manner which optimize the expected utility of the responses given the goal of estimating the model parameters (Lindley, 1956; Bernardo, 1979; MacKay, 1992). Mathematically, the optimization problem is to find at each iteration

(6) $$\begin{array}{c} x_{n+1}^{*}=\underset{x\in X}{\arg\max}\,U_{n}^{\left(E\right)}\left(x\right), \end{array}$$

where $U_{n}^{\left(E\right)}\left(X\right)$ is the estimation utility function based on the data of the first n stimulus–response pairs. There are many choices for this function, including expected squared error (Müller and Parmigiani, 1995), expected prediction error (Sugiyama, 2006), and mutual information between stimuli and model parameters (Paninski, 2005). The generic name for this strategy is *optimal experimental design* or OED (Federov, 1972; Atkinson and Donev, 1992; Cohn et al., 1996), and it is often studied in a Bayesian framework (MacKay, 1992; Chaloner and Verdinelli, 1995). Recent theoretical and experimental work has shown that such methods can potentially be fruitfully applied in neuroscientific experiments (Paninski, 2005; Paninski et al., 2007; Lewi et al., 2009, 2011; DiMattina and Zhang, 2011). Not only can optimal experimental design make the estimation of high-dimensional models practical (Lewi et al., 2009), but can also make it tractable to estimate highly non-linear models which cannot be readily identified from random “white noise” data of the kind typically used in system identification experiments (DiMattina and Zhang, 2010, 2011). We first discuss the application of such methods in psychology and cognitive science, and then discuss recent theoretical and experimental work on applications of OED methods to sensory neurophysiology experiments

### ADAPTIVE STIMULUS OPTIMIZATION IN PSYCHOLOGY AND COGNITIVE SCIENCE

Psychophysics has long utilized adaptive data collection, with the classic example being the staircase method for threshold estimation (Cornsweet, 1962). More recently, an adaptive Bayesian approach to threshold estimation (QUEST) which chooses new stimuli for each trial at the current Bayesian estimate of the threshold was developed (Watson and Pelli, 1983), and subsequent work extended this approach to permit simultaneous estimation of both the threshold and slope of the psychometric function (Snoeren and Puts, 1997). Another line of work applied an information-theoretic approach to estimating the slope and threshold parameters, where stimuli were chosen at each trial to maximize the expected information gained about the slope and threshold parameters (Kontsevich and Tyler, 1999). More sophisticated methods of this kind have been utilized for psychometric functions defined on two-dimensional stimuli (Kujala and Lukka, 2006), with these procedures being applied for estimating contrast sensitivity functions (Lesmes et al., 2010) and color sensitivity of human observers (Kujala and Lukka, 2006). In addition to finding widespread application in sensory psychophysics, adaptive methods have also been used more broadly in the cognitive sciences (Wixted and Ebbesen, 1991; Rubin and Wenzel, 1996; Nosofsky and Zaki, 2002; Opfer and Siegler, 2007; Kujala et al., 2010; McCullough et al., 2010).

### GENERALIZED LINEAR MODELS AND BIOPHYSICAL MODELS

More recently, investigators in computational neuroscience have demonstrated that adaptive information-theoretic sampling where stimuli are chosen to maximize the expected information gain between a stimulus and the model parameters can be a highly effective means of estimating the parameters of sensory processing models (Paninski, 2005; Paninski et al., 2007). A fast information-theoretic algorithm has been developed for the generalized linear model which applies a static non-linearity to the output (Lewi et al., 2009). The generalized linear model has been utilized in numerous studies (Simoncelli et al., 2004) and enjoys a likelihood function with no local maxima (Paninski, 2004). Their algorithm relied on a Gaussian approximation to the posterior density, permitting fast recursive updates, with the calculations for finding the optimal stimulus growing only as the square of the stimulus space dimensionality. Numerical simulations demonstrated that their procedure was asymptotically efficient, with the empirically computed variance of the posterior density converging to the minimum theoretically possible variance.

One issue which potentially affects studies of stimulus optimization is neuronal adaptation due to the stimulus history (Ulanovsky et al., 2003; Asari and Zador, 2009). In sensory neurons, this may be manifested as the system actually changing its underlying parameters which we seek to estimate as the experiment progresses. However, the procedure developed by Lewi et al. (2009) was demonstrated to be robust to parameter drift in numerical simulations, suggesting the ability to compensate for changes to the system brought about by adaptation effects. Furthermore, their model also permits the estimation of a spike-history filter, allowing neuronal response history to influence predictions to new stimuli.

A further study by this group applied this algorithm to fitting generalized linear models to avian auditory neurons probed with conspecific song samples, and it was found that accurate estimation could be obtained using vastly fewer samples when they were chosen adaptively using the algorithm then when they were chosen non-adaptively (Lewi et al., 2011). Although this procedure has yet to be applied in real on-line experiments, it provides experimenters working on a variety of systems with a powerful tool for quickly characterizing neurons whose responses are well described by generalized linear models (Chichilnisky, 2001) or related models (Pillow et al., 2008).

More recently, this group has also applied optimal experimental design to the cellular neuroscience problem of accurately estimating voltages from dendritic trees using measurements suffering from low signal-to-noise ratio (Huggins and Paninski, 2012). Using simulated compartmental models, these authors demonstrated that by adaptively choosing observation locations which minimize the expected squared error of the voltage measurement, a substantial improvement in accuracy was obtained compared to random sampling. This procedure is potentially of great experimental usefulness because techniques like two-photon imaging permit spatially complete observations of dendrites, but with low signal-to-noise ratios (Djurisic et al., 2008; Canepari et al., 2010).

### MULTIPLE LAYER NEURAL NETWORKS

Since many sensory neurons are non-linear (Young et al., 2005; Wu et al., 2006), it is of interest to characterize neurons using various non-linear models, including quadratic and bilinear models (Yu and Young, 2000; Berkes and Wiskott, 2006; Ahrens et al., 2008a,b), neural networks (Lau et al., 2002; Prenger et al., 2004; Cadieu et al., 2007) and basis function networks (Poggio and Girosi, 1990). A generalized linear model is also a non-linear model because it employs a static non-linearity at the output stage. Although a generalized linear model allows limited non-linearities, it enjoys tractable and consistent estimation procedures without problems of local minima (Paninski, 2004). Identifying more complex non-linear models like hierarchical neural networks from physiological data tends to be harder due to problems like local minima and plateaus in the error surface (Amari et al., 2006; Cousseau et al., 2008; Wei and Amari, 2008; Wei et al., 2008).

**FIGURE 4:**
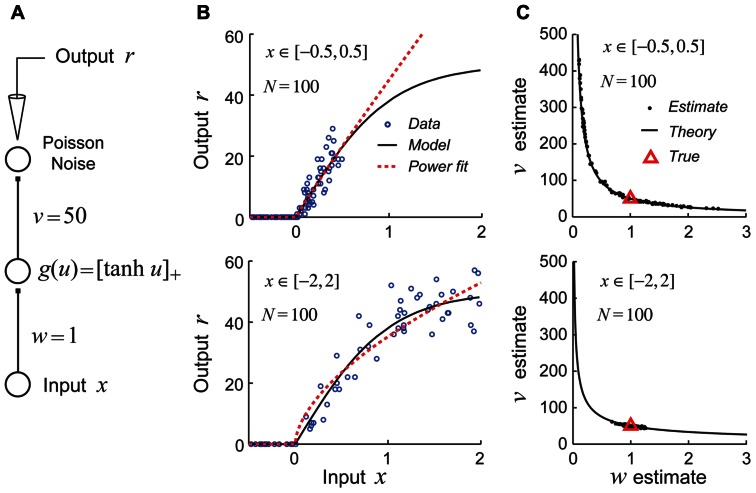
**Example of continuous parameter confounding in a simple non-linear neural network model**. Adapted with permission from DiMattina and Zhang (2010). **(A)** A simple three layer neural network whose input and output weight parameters (*w*,*v*) we wish to estimate from noisy stimulus–response data. Noise is drawn from a Poisson distribution. **(B)** *Top*: The input stimuli *x* ∈ [-0.5, 0.5] only drive the hidden unit over a limited region of its gain function (black curve) which may be well approximated by a power law function (red dashed line). *Bottom*: The input stimuli *x* ∈ [-2,2] drive the hidden unit over a larger region of its gain function which is poorly approximated by a power law function. **(C)** *Top*: When trained with sets of stimuli like that in the top of **Figure 4B**, the estimates (black dots) lie scattered along the curve predicted by the power law confounding theory. *Bottom*: When trained with sets of stimuli like those in the bottom panel of **Figure 4B**, the true parameter values (red triangle) are more reliably recovered.

For studies aimed at estimating generalized linear models, the use of a fixed white-noise stimulus set is often quite effective and is theoretically well-justified (Chichilnisky, 2001; Paninski, 2004; Wu et al., 2006). However, recent theoretical work suggests that using fixed stimulus sets like white noise may be deeply problematic for efforts to identify non-linear hierarchical network models due to *continuous parameter confounding *(DiMattina and Zhang, 2010). This problem is illustrated for a very simple non-linear neural network model shown in **Figure 4A**. In this example, the goal is to recover the parameters (*w,v*) of the network by performing maximum likelihood (ML) estimation given noisy stimulus–response observations. When the input stimuli *x* only drive the hidden unit over a region of its gain function which can be well approximated by a power function (**Figure 4B**, top), the estimates obtained by ML for different datasets lie scattered along the continuum *vw*^α^ = *C*, as one would expect for a power law gain function *g*(*u*) = *Au*^α^ (**Figure 4C**, top). (Here the constant $C=v_{T}w_{T}^{\alpha }$, where *w*_T_ and *v*_T_ are the true values of the input and output weights.) In contrast, when the input stimuli *x* drive the hidden unit over a full range of its gain so that the power law approximation is poor (**Figure 4B**, bottom), the true parameters are accurately recovered for different datasets (**Figure 4C**, bottom).

**FIGURE 5:**
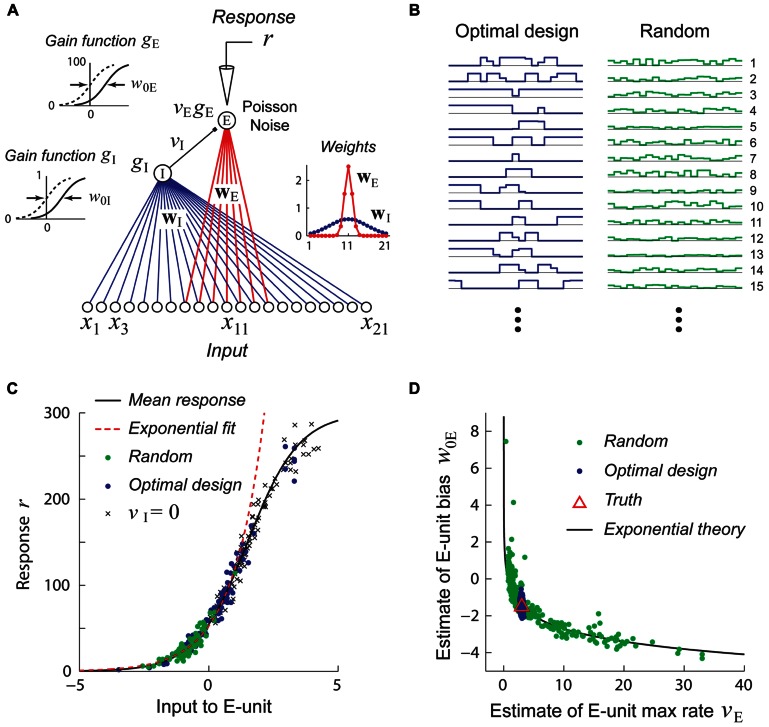
**Stimuli which are adaptively optimized for accurate parameter estimation can be more effective than random stimuli for recovering non-linear models**. Adapted with permission from DiMattina and Zhang (2011). **(A)** A simple center-surround neural network consisting of a narrowly integrating excitatory output unit (E-unit) which receives inhibitory input from a broadly integrating interneuron (I-unit). **(B)** Examples of optimally designed (left) and random (right) stimuli. Note that the optimally designed stimuli exhibit complex correlated structure. **(C)** Random stimuli (green dots) only drive the E-unit over a limited range of its gain function (black curve) which may be well approximated by an exponential function (red dashed line). This is due to inhibition from the I-unit, as can be seen by setting *v*_I_ = 0 (crosses). By contrast, optimally designed stimuli (blue dots) drive the gain function over its full range. **(D)** Estimates attained from training with random stimuli (green dots) exhibit continuous parameter confounding between the output weight and bias, as predicted by the exponential theory (black curve). In contrast, estimates attained from optimally designed stimuli accurately recover the true parameters (red triangle).

A hypothetical experiment which suffers from this problem is illustrated in **Figure 5**. We see that when the network in **Figure 5A** is probed with random stimuli (**Figure 5B**, right), the hidden unit is driven over a limited range of its gain function which may be well approximated by an exponential, so that the sigmoidal gain (**Figure 5C**, black curve) may *de facto* be replaced by a new exponential gain function *g*(*u*) = *Ae*^α^^*u*^ (**Figure 5C**, red curve). With this new gain, it follows that a continuum of different values of the output weight *v* and bias *w*_0_ lying on the curve *ve*^α^^*w*_0_^ = *C* will yield models whose responses to the training data are indistinguishable and therefore multiple estimates of these parameters from different random training sets will lie scattered along this curve (**Figure 5D**). (Here the constant *C* = *v*_T_e^α^*w*_0T_ where *V*_T_ and *w*_0T_ are the true values of the output weight and hidden unit bias.)

Adaptive stimulus optimization methods like information-theoretic sampling (Paninski, 2005) can in principle overcome this problem of continuous parameter confounding, as we see in **Figure 5D** where the correct network parameters are reliably recovered when optimally designed stimuli (**Figure 5B**, left) are used. This simple example suggests that adaptive stimulus optimization may make it tractable to reliably recover the parameters of complex hierarchical networks needed to model non-linear neurons, whereas it is much harder to recover these networks using standard stimulus sets like white noise.

Many previous studies in the statistics and machine learning literature have demonstrated that faster convergence and smaller generalization error may be obtained when neural networks are trained adaptively using optimally designed stimuli (Lindley, 1956; MacKay, 1992; Cohn et al., 1994). Recently, we have developed a practical method for implementing the information-theoretic stimulus optimization approach derived for generalized linear models (Lewi et al., 2009) for arbitrary non-linear models like hierarchical neural networks. Although this method employs numerous approximations, it has been shown in simulated experiments to be effective at recovering non-linear neural networks having multiple hidden units, and is fast enough to utilize in real experiments (Tam et al., 2011; Dekel, 2012; Tam, 2012).

## ADAPTIVE OPTIMIZATION FOR SENSORY MODEL COMPARISON

Quite often the appropriate model for describing a sensory neuron or perceptual quantity is unknown. Therefore, an important experimental goal may be to discriminate between two or more competing models. Mathematically, the optimization problem is to iteratively find stimuli

(7) $$\begin{array}{c} x_{n+1}^{*}=\underset{x}{\arg\max}\,U_{n}^{\left(C\right)}\left(x\right), \end{array}$$

which optimize a model comparison utility function *U*_n_^(C)^ (**x**), one choice of which may be the expected reduction in model space entropy (Cavagnaro et al., 2010; DiMattina and Zhang, 2011). This equation may be regarded as the optimal comparison counterpart of the equation for optimal estimation (Eq. 6). We now briefly discuss recent studies making use of adaptive stimulus optimization for model selection.

### PSYCHOPHYSICAL MODEL COMPARISON

Although standard model comparison methods like the Bayesian Information Criterion (BIC; Schwarz, 1978) or predictive cross-validation may be applied *post hoc* (Vladusich et al., 2006; Wu et al., 2006), numerous studies suggest that performing experiments using stimuli optimized for model comparison may be far more effective (Atkinson and Fedorov, 1975a,b). One method for model comparison developed recently for psychophysical experiments is known as MAximum Differentiation (MAD) competition (Wang and Simoncelli, 2008). Given two perceptual models which relate stimulus parameters to a perceptual quantity, this method generates a pair of stimuli which maximizes/minimizes the response of one model while holding the other model’s response fixed. Next, this procedure is repeated with the role of the two models reversed. Testing human subjects on the two pairs of synthesized stimuli can determine which model is “better” in the sense of telling us which model’s max/min pairs are simpler to discriminate. This procedure has been fruitfully applied to comparing image quality assessment models which aim to predict human perception of image quality (Wang and Bovik, 2006)

An information-theoretic method for model comparison was recently derived by Cavagnaro et al. (2010). Given a set of models with the *i*-th model having prior probability *P*_0_(*i*), stimuli are chosen to maximize the mutual information between the stimulus and the model index *i* by minimizing the expected model space entropy in a manner directly analogous to information-theoretic model estimation (Paninski, 2005), except that in this case the unknown variable is a discrete model index *i* rather than a continuous parameter value **θ**. This method was applied to competing models of memory retention from the cognitive science literature (Rubin et al., 1999) and was shown to permit much more accurate discrimination than standard non-adaptive methods.

### NEURAL MODEL COMPARISON

In general, the correct parameters of competing sensory processing models are not known beforehand. Therefore, it is of interest to consider how to conduct experiments which estimate and discriminate competing models. Typically, investigators in neurophysiology and neuroimaging have applied model-comparison techniques *post hoc* (David and Gallant, 2005; Vladusich et al., 2006; Penny, 2012), particularly in the system identification literature (Prenger et al., 2004; David and Gallant, 2005; Wu et al., 2006; Sharpee et al., 2008; Rabinowitz et al., 2012; Schinkel-Bielefeld et al., 2012). However, a fundamental limitation with *post hoc* analysis is that it is not possible to generate and test critical stimuli which are optimized for model comparison, as this is only possible while the system is under observation. This limitation can only be overcome by fitting multiple models to a sensory neuron during the course of an experiment and then using the fitted models to generate and present critical stimuli which are optimized to best discriminate the models. Although previous work has presented stimuli on-line to test or verify a single model (deCharms et al., 1998; Touryan et al., 2002), very little work in single-unit *in vivo* sensory neurophysiology has presented stimuli optimized for model comparison in real-time (Tam et al., 2011).

**FIGURE 6:**
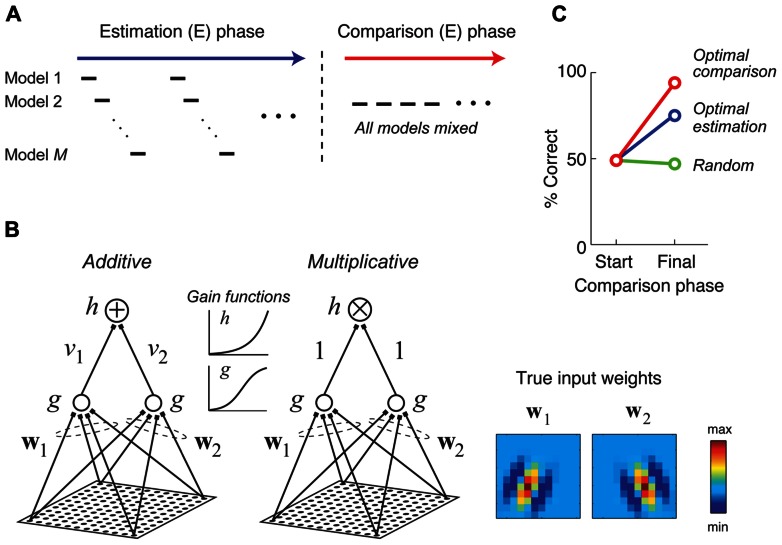
**Stimuli which are adaptively optimized for model comparison can lead to more accurate model selection**. Adapted with permission from DiMattina and Zhang (2011). **(A)** A hypothetical two phase procedure for estimating and comparing multiple competing models. During the estimation (E) phase, stimuli are optimized in turn for estimating each model. During the comparison (C) phase, stimuli are optimized for comparing all of the models. **(B)** Two candidate models were fit to data generated by a true additive model whose input weights (w_1_ and w_2_) were 12 × 12 Gabor patches shown at the right. The two competing models differ only in their method of integrating subunit activities (additive versus multiplicative). **(C)** At the end of the estimation phase (“Start”), the BIC does not consistently prefer either model. Presenting additional stimuli optimized for model discrimination yields almost perfect model selection (red curve), while presenting additional random stimuli (green curve), or stimuli optimized for model estimation (blue curve) either does not improve or only somewhat improves model selection.

A recent study considered a two-stage approach for combining the goals of model estimation and comparison in neurophysiology experiments, illustrated schematically in **Figure 6A** (DiMattina and Zhang, 2011). In the first stage, stimuli are adaptively optimized for parameter estimation, with the optimal stimulus for each model being presented in turn. In the second stage, stimuli are generated adaptively in order to optimally discriminate competing models making use of an information-theoretic criterion (Cavagnaro et al., 2010) or a likelihood-based criterion. In the special case of two models *f*_1_ (**x**, **θ**_1_), *f*_2_ (**x**, **θ**_2_) and Gaussian response noise, it can be shown that under a likelihood criterion the best stimulus for model discrimination is the stimulus which maximizes the quantity [*f*_1_ (**x**, **θ**_1_) - *f*_2_ (**x**, **θ**_2_)]^2^, and furthermore this stimulus will maximally increase the BIC in favor of the best model (DiMattina and Zhang, 2011).

**Figure 6** illustrates a numerical experiment making use of this two-stage procedure for the problem of discriminating an additive and multiplicative model of neural responses (**Figure 6B**), where the additive model is assumed to be the true model. After the estimation phase, the BIC does not have a strong preference for either model, only being correct about half the time (**Figure 6C**). However, after presenting 500 stimuli optimized for discriminating the additive and multiplicative model and applying the BIC to all available data, the correct (additive) model is preferred for 24 of 25 Monte Carlo trials (red curve). As a control, presenting additional stimuli optimized for model estimation only improves final model selection moderately (blue curve), while presenting random stimuli does not at all improve model selection performance (green curve). This procedure has now been applied in neurophysiology experiments to generate critical stimuli to distinguish between two competing models of spectral processing by single neurons in the primate inferior colliculus (Tam et al., 2011; Tam, 2012).

## DISCUSSION

**FIGURE 7:**
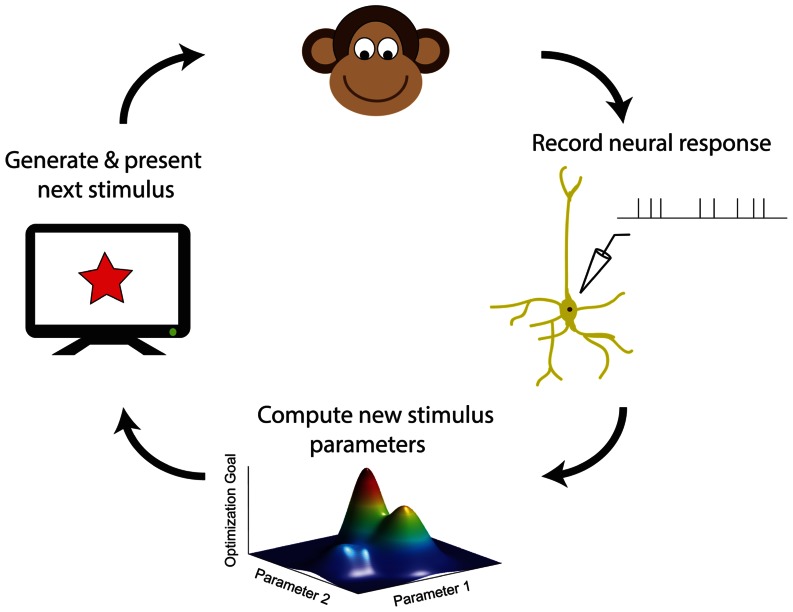
**A schematic summary of closed-loop approaches in sensory neurophysiology**. Neural responses to previous stimuli are used in order to choose new stimuli, by maximizing an objective function for accomplishing a desired experimental goal (see also **Table 1**).

With increasing computer power, it is becoming practical for neuroscience experiments to utilize adaptive stimulus optimization where stimuli are generated in real-time during the course of the experiment (Benda et al., 2007; Newman et al., 2013). Various experiments have utilized adaptive stimulus optimization in order to break the “curse of dimensionality” and find the optimal stimulus for a sensory neuron in spaces which are too large for factorial exploration (O’Connor et al., 2005; Yamane et al., 2008). However, simply characterizing the optimal stimulus for a sensory neuron provides at best only a partial description of neural coding (Olshausen and Field, 2005). Therefore, in addition to helping to find the optimal stimulus, adaptive stimulus optimization makes it possible to pursue engineering-inspired approaches to sensory neurophysiology which may yield greater functional insights, for instance finding stimulus ensembles maximizing information between stimuli (Machens, 2002; Machens et al., 2005) and neural responses or fitting and comparing multiple non-linear models to neural responses (Lewi et al., 2009; DiMattina and Zhang, 2011). **Table 1** summarizes the various closed-loop stimulus optimization paradigms discussed in this review, and **Figure 7** schematically illustrates the closed-loop experimental approach.

**Table 1:** Summary of various closed-loop stimulus optimization approaches utilized in sensory systems neuroscience.

Optimization goal	Equation	Example references
Firing rate optimization	1	Nelken et al. (1994); O’Connor et al. (2005), Yamane et al. (2008); Koelling and Nykamp (2012)
Iso-response surfaces	4	Gollisch et al. (2002); Bölinger and Gollisch (2012), Horwitz and Hass (2012)
Maximally informative stimulus ensembles	5	Machens (2002); Machens et al. (2005)
On-line model estimation	6	Lewi et al. (2009),^2011^, DiMattina and Zhang (2011)
On-line model comparison	7	Cavagnaro et al. (2010); DiMattina and Zhang (2011)

The vast majority of the work to date has applied closed-loop methods to studying scalar firing rate responses measured from single neurons. However, as closed-loop approaches are continuing to develop, and as new techniques like optical imaging (Ohki et al., 2005; Bock et al., 2011) are making it increasingly feasible to observe large numbers of neurons simultaneously, it is of great interest for future investigations to apply these methods to neural populations and to measurements beyond scalar firing rate. Here we briefly discuss some possible directions for future research.

While the notion of the optimal stimulus is well-defined for single neurons, it is not well-defined for neural populations. However, an alternative approach to stimulus optimization for a population of neurons is to find the stimulus at which the population is best at discriminating nearby stimuli, as opposed to the stimulus yielding the highest firing rate response. Indeed, it has been suggested by a number of investigators that high-slope regions of tuning curves, where nearby stimuli are best discriminated, are much more important in sensory coding than tuning curve peaks (Seung and Sompolinsky, 1993; Harper and McAlpine, 2004; Butts and Goldman, 2006; Bonnasse-Gahot and Nadal, 2008). Under reasonable assumptions of independent Poisson responses, the one-dimensional stimulus *x* at which a neural population can best discriminate nearby stimuli *x* + δ*x* is the stimulus which maximizes the Fisher information $I_{F}\left(x\right)=\Sigma_{i=1}^{N}{\left[f_{i}^{\prime }\left(x\right)\right]}^{2}/fi\left(x\right)$, where *f*_*i*_(*x*) is the tuning curve of the *i*-th neuron (Dayan et al., 2001). It is relatively straightforward to extend this Fisher information formalism to higher dimensional stimulus spaces (Zhang and Sejnowski, 1999; Johnson et al., 2001; Bethge et al., 2002). Local approximation of the Fisher information matrix has been used in previous work aimed at stimulus optimization in a single neuron (Bandyopadhyay et al., 2007b), and this technique could readily generalize to find the stimulus which is best discriminated from nearby stimuli by a population code.

Extension of the definition of iso-response surfaces (Gollisch et al., 2002) to multiple neurons is relatively straightforward. In particular, if we can view each neuron as implementing a function *f*(**x**) on the stimulus space, then the region of stimulus space which simultaneously satisfies multiple constraints *f*_1_ (**x**) = *c*_1_, ·, *f*_*N*_(**x**) = *c_N_* should simply be the (possibly empty) intersection of the regions of stimulus space satisfying each individual constraint. It would be interesting to extend the maximally informative ensemble approach (Machens, 2002) to multiple neurons as well. One potential difficulty is that the number of possible responses which one needs to measure to compute the probability distribution *p*(y | **x**) increases exponentially with the number of neurons in the population. Indeed, this exponential increase in the number of symbols with the dimensionality of the response space is a well-known problem with applications of information-theoretic methods in neuroscience (Rieke et al., 1997). It would be desirable to develop more efficient computational techniques for studying neuronal populations in the future (Yarrow et al., 2012).

In addition to considering neural populations, another direction for extending the closed-loop paradigm is to consider neural responses more sophisticated than firing rates, for instance the temporal patterns of neural responses (Optican and Richmond, 1987; Victor and Purpura, 1996), first spike latency (VanRullen et al., 2005; Gollisch and Meister, 2008), or synchronous responses in neural populations (Brette, 2012). Since a temporal pattern is a vector but not a scalar, one needs to extract a scalar quantity from a temporal pattern in order to define the optimal stimulus. For example, synchrony can be defined as a scalar quantity (Steinmetz et al., 2000) and can in principle be optimized over a stimulus space in the same manner as firing rate. The iso-response paradigm (Gollisch et al., 2002) would generalize quite well to both spike pattern and synchrony measures. In this case of spike pattern, the goal would be to find the equivalence class of all stimuli which could elicit a desired pattern of spiking, and theoretical efforts have demonstrated that it is possible to design stimuli to produce a desired spike pattern (Ahmadian et al., 2011). Similarly, for iso-synchrony curves one could find equivalence classes of stimuli yielding the same degree of synchrony in the population by utilizing algorithms similar to those developed for firing rate.

One of the most powerful applications of the closed-loop paradigm is the ability to move sensory neurophysiology toward a model-based paradigm, where experiments are performed with the goal of identifying and comparing multiple competing non-linear models (Paninski, 2005; Lewi et al., 2009; DiMattina and Zhang, 2011; Tam et al., 2011). One advantage of model identification is that successful identification gives the experimenter a variety of biologically important information about the neuron or neuronal population “for free.” That is, once one has determined an accurate model for a sensory neuron, the optimal stimulus for maximizing firing rate, the iso-response surfaces, or the stimulus ensemble maximizing information transmission can be predicted from this model, and these predictions can be tested experimentally. However, the model-based approach is not without its difficulties, as many sensory neurons are poorly described by tractable linear and quadratic models and may be better described by more complex models like basis functions and neural networks. Recent work has demonstrated that in principle, adaptive stimulus optimization methods long utilized in machine learning and psychophysics can be applied in sensory neurophysiology for purposes of model estimation and comparison (Paninski, 2005; Lewi et al., 2009; DiMattina and Zhang, 2011). In particular, our recent study has presented a practical two-stage experimental method for generating stimuli which are optimal for estimating the parameters of multiple non-linear models and then generating stimuli on-line in order to critically compare the predictions of different models (DiMattina and Zhang, 2011). This method is presently being applied in ongoing auditory neurophysiology studies (Tam et al., 2011; Dekel, 2012; Tam, 2012), and may be applicable to a broad variety of sensory systems.

## Conflict of Interest Statement

The authors declare that the research was conducted in the absence of any commercial or financial relationships that could be construed as a potential conflict of interest.

## References

[B1] Ahmadian Y. Packer A. M. Yuste R. Paninski L. (2011). Designing optimal stimuli to control neuronal spike timing. *J. Neurophysiol.* 106 1038–1053 21511704 10.1152/jn.00427.2010 PMC3154808

[B2] Ahrens M. B. Linden J. F. Sahani M. (2008a). Nonlinearities and contextual influences in auditory cortical responses modeled with multilinear spectrotemporal methods. *J. Neurosci*. 28 1929–1942 18287509 10.1523/JNEUROSCI.3377-07.2008 PMC6671443

[B3] Ahrens M. B. Paninski L. Sahani M. (2008b). Inferring input nonlinearities in neural encoding models. *Network* 19 35–67 18300178 10.1080/09548980701813936

[B4] Albrecht D. G. DeValois R. L. Thorell L. G. (1980). Visual cortical neurons: are bars or gratings the optimal stimuli? *Science* 207 88–90 6765993 10.1126/science.6765993

[B5] Albrecht D. G. Hamilton D. B. (1982). Striate cortex of monkey and cat: contrast response function. *J Neurophysiol*. 48 217–237 7119846 10.1152/jn.1982.48.1.217

[B6] Amari S.-I. Park H. Ozeki T. (2006). Singularities affect dynamics of learning in neuromanifolds. *Neural Comput*. 18 1007–1065 16595057 10.1162/089976606776241002

[B7] Anderson M. J. Micheli-Tzanakou E. (2002). Auditory stimulus optimization with feedback from fuzzy clustering of neuronal responses. *IEEE Trans. Inf. Technol. Biomed*. 6 159–170 12075670 10.1109/titb.2002.1006303

[B8] Arimoto S. (1972). An algorithm for computing the capacity of arbitrary discrete memoryless channels. *IEEE Trans. Inf. Theory* 18 14–20

[B9] Asari H. Zador A. M. (2009). Long-lasting context dependence constrains neural encoding models in rodent auditory cortex. *J. Neurophysiol.* 102 2638–2656 19675288 10.1152/jn.00577.2009 PMC2777827

[B10] Atkinson A. Fedorov V. (1975a). The design of experiments for discriminating between two rival models. *Biometrika* 62 57–70

[B11] Atkinson A. Fedorov V. (1975b). Optimal design: experiments for discriminating between several models. *Biometrika* 62 289–303

[B12] Atkinson A. C. Donev A. N. (1992). *Optimum Experimental Designs*. Oxford: Clarendon Press

[B13] Bandyopadhyay S. Reiss L. A. J. Young E. D. (2007a). Receptive field for dorsal cochlear nucleus neurons at multiple sound levels. *J. Neurophysiol.* 98 3505–3515 17898144 10.1152/jn.00539.2007

[B14] Bandyopadhyay S. Young E. D Reiss L. A. J. (2007b). “Spectral edges as optimal stimuli for the dorsal cochlear nucleus,” in *Hearing – From Basic Research to Applications* eds Kollmeier B. Klump G. Hohmann V. Langemann U. Mauermann M. Upperkamp S. (New York: Springer-Verlag) 39–45

[B15] Barbour D. L. Wang X. (2003a). Auditory cortical responses elicited in awake primates by random spectrum stimuli. *J. Neurosci.* 23 7194–7206 12904480 10.1523/JNEUROSCI.23-18-07194.2003 PMC1945239

[B16] Barbour D. L. Wang X. (2003b). Contrast tuning in auditory cortex. *Science* 299 1073–1075 12586943 10.1126/science.1080425 PMC1868436

[B17] Barlow H. B. (1961). “Possible principles underlying the transformation of sensory messages,” in *Sensory Communication* ed Rosenblith W. A. (Cambridge, MA: MIT Press) 217–234

[B18] Bartlett E. L. Wang X. (2005). Long-lasting modulation by stimulus context in primate auditory cortex. *J. Neurophysiol.* 94 83–104 15772236 10.1152/jn.01124.2004

[B19] Bellman R. (1961). *Adaptive Control Processes: A Guided Tour*. Princeton, NJ: Princeton University Press

[B20] Benda J. Gollisch T. Machens C. K. Herz A. V. (2007). From response to stimulus: adaptive sampling in sensory physiology. *Curr. Opin. Neurobiol.* 17 430–436 17689952 10.1016/j.conb.2007.07.009

[B21] Berkes P. Wiskott L. (2005). Slow feature analysis yields a rich repertoire of complex cell properties. *J. Vis.* 5 579–602 16097870 10.1167/5.6.9

[B22] Berkes P. Wiskott L. (2006). On the analysis and interpretation of inhomogeneous quadratic forms as receptive fields. *Neural Comput.* 18 1868–1895 16771656 10.1162/neco.2006.18.8.1868

[B23] Berkes P. Wiskott L. (2007). Analysis and interpretation of quadratic models of receptive fields. *Nat. Protoc.* 2 400–407 17406601 10.1038/nprot.2007.27

[B24] Bernardo J. (1979). Expected information as expected utility. *Ann. Stat.* 7 686–690

[B25] Bethge M. Rotermund D. Pawelzik K. (2002). Optimal short-term population coding: when fisher information fails. *Neural Comput.* 14 2317–2751 12396565 10.1162/08997660260293247

[B26] Blahut R. (1972). Computation of channel capacity and rate-distortion functions. *IEEE Trans. Inf. Theory* 18 460–473

[B27] Bleeck S. Patterson R. D. Winter I. M. (2003). Using genetic algorithms to find the most effective stimulus for sensory neurons. *J. Neurosci. Methods* 125 73–82 12763233 10.1016/s0165-0270(03)00040-2

[B28] Bock D. D. Lee W.-C. A. Kerlin A. M. Andermann M. L. Hood G. Wetzel A. W. (2011). Network anatomy and in vivo physiology of visual cortical neurons. *Nature*, 471 177–182 21390124 10.1038/nature09802 PMC3095821

[B29] Bölinger D. Gollisch T. (2012). Closed-loop measurements of iso-response stimuli reveal dynamic nonlinear stimulus integration in the retina. *Neuron* 73 333–346 22284187 10.1016/j.neuron.2011.10.039

[B30] Bonnasse-Gahot L. Nadal J. P. (2008). Neural coding of categories: information efficiency and optimal population codes. *J. Comput. Neurosci.* 25 169–187 18236147 10.1007/s10827-007-0071-5

[B31] Brette R. (2012). Computing with neural synchrony. *PLoS Comput. Biol* 8: e1002561 10.1371/journal.pcbi.1002561 PMC3375225 22719243

[B32] Butts D. A. Goldman M. S. (2006). Tuning curves, neuronal variability, and sensory coding. *PLoS Biol.* 4: e92 10.1371/journal.pbio.0040092 PMC1403159 16529529

[B33] Cadieu C. Kouh M. Pasupathy A. Connor C. E. Riesenhuber M. Poggio T. (2007). A model of v4 shape selectivity and invariance. *J. Neurophysiol.* 98 1733–1750 17596412 10.1152/jn.01265.2006

[B34] Canepari M. Willadt S. Zecevic D. Vogt K. (2010). Imaging inhibitory synaptic potentials using voltage sensitive dyes. *Biophys. J.* 98 2032–2040 20441768 10.1016/j.bpj.2010.01.024 PMC2862202

[B35] Carlson E. T. Tasquinha R. J. Zhang K. Connor C. E. (2011). A sparse object coding scheme in area v4. *Curr. Biol.* 21 288–293 21315595 10.1016/j.cub.2011.01.013 PMC3070463

[B36] Cavagnaro D. R. Myung J. I. Pitt M. A. Kujala J. V. (2010). Adaptive design optimization: a mutual information-based approach to model discrimination in cognitive science. *Neural Comput.* 22 887–905 20028226 10.1162/neco.2009.02-09-959

[B37] Chaloner K. Verdinelli I. (1995). Bayesian experimental design: a review. *Stat. Sci.* 10 273–304

[B38] Chambers A. Hancock K. E. Polley D. B. (2012). An adaptive stimulus search method for rapid characterization of multidimensional receptive fields in auditory cortex of awake animals. *Soc. Neurosci. Abstr.* 460 15

[B39] Chase S. M. Young E. D. (2008). Cues for sound localization are encoded in multiple aspects of spike trains in the inferior colliculus. *J. Neurophysiol.* 99 1672–1682 18234986 10.1152/jn.00644.2007

[B40] Cheng K. Hasegawa T. Saleem K. S. Tanaka K. (1994). Comparison of neuronal selectivity for stimulus speed, length, and contrast in the prestriate visual cortical areas v4 and mt of the macaque monkey. *J. Neurophysiol.* 71 2269–2280 7931516 10.1152/jn.1994.71.6.2269

[B41] Chichilnisky E. J. (2001). A simple white noise analysis of neuronal light responses. *Network* 12 199–213 11405422

[B42] Cohn D. Atlas L. Ladner R. (1994). Improving generalization with active learning. *Mach. Learn.* 15 201–221

[B43] Cohn D. A. Ghahramani Z. Jordan M. I. (1996). Active learning with statistical models. *J. Artif. Intell. Res.* 4 129–145

[B44] Cornsweet T. N. (1962). The staircase method in psychophysics. *Am. J. Psychol.* 75 485–491 13881416

[B45] Cousseau F. Ozeki T. Amari S.-I. (2008). Dynamics of learning in multilayer perceptrons near singularities. *IEEE Trans. Neural Netw.* 19 1313–1328 18701364 10.1109/TNN.2008.2000391

[B46] David S. Gallant J. (2005). Predicting neuronal responses during natural vision. *Network* 16 239–260 16411498 10.1080/09548980500464030

[B47] David S. Mesgarani N. Fritz J. Shamma S. (2009). Rapid synaptic depression explains nonlinear modulation of spectro-temporal tuning in primary auditory cortex by natural stimuli. *J. Neurosci.* 29 3374–3386 19295144 10.1523/JNEUROSCI.5249-08.2009 PMC2774136

[B48] Dayan P. Abbott L. F. Abbott L. (2001). *Theoretical Neuroscience: Computational and Mathematical Modeling of Neural Systems*. Cambridge, MA: MIT Press

[B49] deCharms R. C. Blake D. Merzenich M. (1998). Optimizing sound features for cortical neurons. *Science* 280 1439–1444 9603734 10.1126/science.280.5368.1439

[B50] Dekel E. (2012). *Adaptive On-line Modeling in the Auditory System: In Vivo Implementation of the Optimal Experimental Design Paradigm*. Master's thesis, Department of Biomedical Engineering, Johns Hopkins University, Baltimore, MD.

[B51] DiMattina C. Wang X. (2006). Virtual vocalization stimuli for investigating neural representations of species-specific vocalizations. *J. Neurophysiol.* 95 1244–1262 16207780 10.1152/jn.00818.2005

[B52] DiMattina C. Zhang K. (2008). How optimal stimuli for sensory neurons are constrained by network architecture. *Neural Comput.* 20 668–708 18045019 10.1162/neco.2007.11-05-076

[B53] DiMattina C. Zhang K. (2010). How to modify a neural network gradually without changing its input–output functionality. *Neural Comput.* 22 1–47 19842986 10.1162/neco.2009.05-08-781

[B54] DiMattina C. Zhang K. (2011). Active data collection for efficient estimation and comparison of nonlinear neural models. *Neural Comput.* 23 2242–2288 21671794 10.1162/NECO_a_00167

[B55] Djurisic M. Popovic M. Carnevale N. Zecevic D. (2008). Functional structure of the mitral cell dendritic tuft in the rat olfactory bulb. *J. Neurosci.* 28 4057–4068 18400905 10.1523/JNEUROSCI.5296-07.2008 PMC6670455

[B56] Douglas S. C. Amari S. Kung S. Y. (2000). On gradient adaptation with unit norm constraints. *IEEE Trans. Signal Proc.* 48 1843–1847

[B57] Federov V. V. (1972). *Theory of Optimal Experiments*. New York: Academic Press

[B58] Felleman D. J Van Essen D. C. (1991). Distributed hierarchical processing in the primate cerebral cortex. *Cereb. Cortex* 1 1–47 1822724 10.1093/cercor/1.1.1-a

[B59] Foldiak P. (2001). Stimulus optimisation in primary visual cortex. *Neurocomputing* 38–40 1217–1222

[B60] Goldberg D. (1989). *Genetic Algorithms in Search, Optimization, and Machine Learning*. Upper Saddle River, NJ: Addison-Wesley Professional

[B61] Gollisch T. Herz A. (2005). Disentangling sub-millisecond processes within an auditory transduction chain. *PLoS Biol.* 3:e8. 10.1371/journal.pbio.0030008 PMC539322 15660161

[B62] Gollisch T. Meister M. (2008). Rapid neural coding in the retina with relative spike latencies. *Science* 319 1108–1111 18292344 10.1126/science.1149639

[B63] Gollisch T. Schütze H. Benda J Herz A. V. M. (2002). Energy integration describes sound-intensity coding in an insect auditory system. *J. Neurosci.* 22 10434–10448 12451143 10.1523/JNEUROSCI.22-23-10434.2002 PMC6758769

[B64] Han X. Xu C. Prince J. (2003). A topology preserving level set method for geometric deformable models. *IEEE Trans. Pattern Anal. Mach. Intell.* 25 755–768

[B65] Harper N. S. McAlpine D. (2004). Optimal neural population coding of an auditory spatial cue. *Nature* 430 682–686 15295602 10.1038/nature02768

[B66] Harth E. Tzanakou E. (1974). Alopex: a stochastic method for determining visual receptive fields. *Vis. Res.* 14 1475–1482 4446379 10.1016/0042-6989(74)90024-8

[B67] Heeger D. J. (1992a). Half-squaring in responses of cat striate cells. *Vis. Neurosci.* 9 427–443 1450099 10.1017/s095252380001124x

[B68] Heeger D. J. (1992b). Normalization of cell responses in cat striate cortex. *Vis. Neurosci.* 9 181–197 1504027 10.1017/s0952523800009640

[B69] Horwitz G. D. Hass C. A. (2012). Nonlinear analysis of macaque v1 color tuning reveals cardinal directions for cortical color processing. *Nat. Neurosci.* 15 913–919 22581184 10.1038/nn.3105 PMC3528341

[B70] Hubel D. H. Wiesel T. N. (1962). Receptive fields, binocular interaction and functional architecture in the cat's visual cortex. *J. Physiol.* 160 106–154 14449617 10.1113/jphysiol.1962.sp006837 PMC1359523

[B71] Huggins J. Paninski L. (2012). Optimal experimental design for sampling voltage on dendritic trees in the low-SNR regime. *J. Comput. Neurosci.* 32 347–366 21861199 10.1007/s10827-011-0357-5

[B72] Hung C. C. Carlson E. T. Connor C. E. (2012). Medial axis shape coding in macaque inferotemporal cortex. *Neuron* 74 1099–1113 22726839 10.1016/j.neuron.2012.04.029 PMC3398814

[B73] Ingham N. McAlpine D. (2004). Spike-frequency adaptation in the inferior colliculus. *J. Neurophysiol.* 91 632–645 14534290 10.1152/jn.00779.2003

[B74] Johnson D. H. Gruner C. M. Baggerly K. Seshagiri C. (2001). Information-theoretic analysis of neural coding. *J. Comput. Neurosci.* 10 47–69 11316339 10.1023/a:1008968010214

[B75] Kennedy J. Eberhart R. (1995). “Particle swarm optimization,” in *Proceedings of the IEEE International Conference on Neural Networks* Vol. 4 (Washington, DC: IEEE) 1942–1948

[B76] Kirkpatrick S. Gelatt C. D. Jr. Vecchi M. P. (1983). Optimization by simulated annealing. *Science* 220 671–680 17813860 10.1126/science.220.4598.671

[B77] Koelling M. E. Nykamp D. Q. (2008). Computing linear approximations to nonlinear neuronal response. *Network* 19 286–313 18991145 10.1080/09548980802503139

[B78] Koelling M. E. Nykamp D. Q. (2012). Searching for optimal stimuli: ascending a neuron's response function. *J. Comput. Neurosci.* 33 449–473 22580579 10.1007/s10827-012-0395-7

[B79] Kontsevich L. L. Tyler C. W. (1999). Bayesian adaptive estimation of psychometric slope and threshold. *Vis. Res.* 39 2729–2737 10492833 10.1016/s0042-6989(98)00285-5

[B80] Kujala J. Lukka T. (2006). Bayesian adaptive estimation: the next dimension. *J. Math. Psychol.* 50 369–389

[B81] Kujala J. Richardson U. Lyytinen H. (2010). A Bayesian-optimal principle for learner-friendly adaptation in learning games. *J. Math. Psychol.* 54 247–255

[B82] Lau B. Stanley G. B. Dan Y. (2002). Computational subunits of visual cortical neurons revealed by artificial neural networks. *Proc. Natl. Acad. Sci. U.S.A.* 99 8974–8979 12060706 10.1073/pnas.122173799 PMC124408

[B83] Lesmes L. A. Lu Z.-L. Baek J. Albright T. D. (2010). Bayesian adaptive estimation of the contrast sensitivity function: the quick CSF method. *J. Vis.* 10 17.1–17.21 20377294 10.1167/10.3.17 PMC4439013

[B84] Lewi J. Butera R. Paninski L. (2009). Sequential optimal design of neurophysiology experiments. *Neural Comput.* 21 619–687 18928364 10.1162/neco.2008.08-07-594

[B85] Lewi J. Schneider D. M. Woolley S. M. N. Paninski L. (2011). Automating the design of informative sequences of sensory stimuli. *J. Comput. Neurosci.* 30 181–200 20556641 10.1007/s10827-010-0248-1 PMC3244976

[B86] Lindley D. (1956). On a measure of the information provided by an experiment. *Ann. Math. Stat.* 27 986–1005

[B87] Machens C. K. (2002). Adaptive sampling by information maximization. *Phys. Rev. Lett.* 88 228104 10.1103/PhysRevLett.88.228104 12059456

[B88] Machens C. K. Gollisch T. Kolesnikova O Herz A. V. M. (2005). Testing the efficiency of sensory coding with optimal stimulus ensembles. *Neuron* 47 447–456 16055067 10.1016/j.neuron.2005.06.015

[B89] MacKay D. (1992). Information-based objective functions for active data selection. *Neural Comput.* 4 590–604

[B90] McCullough M. E. Luna L. R. Berry J. W. Tabak B. A. Bono G. (2010). On the form and function of forgiving: modeling the time-forgiveness relationship and testing the valuable relationships hypothesis. *Emotion* 10 358–376 20515225 10.1037/a0019349

[B91] Müller P. Parmigiani G. (1995). Optimal design *via* curve fitting of Monte Carlo experiments. *J. Am. Stat. Assoc.* 90 1322–1330

[B92] Nelder J. Mead R. (1965). A simplex method for function minimization. *Comput. J.* 7 308–313

[B93] Nelken I. Prut Y. Vaadia E. Abeles M. (1994). In search of the best stimulus: an optimization procedure for finding efficient stimuli in the cat auditory cortex. *Hear. Res.* 72 237–253 8150740 10.1016/0378-5955(94)90222-4

[B94] Newman J. Zeller-Townson R. Fong M. Desai S. Gross R. Potter S. (2013). Closed-loop, multichannel experimentation using the open-source neurorighter electrophysiology platform. *Front. Neural Circuits* 6: 98 10.3389/fncir.2012.00098 PMC3548271 23346047

[B95] Nocedal J. Wright S. (2006). *Numerical Optimization*. *Springer Series in Operations Research and Financial Engineering* 2 Edn. New York: Springer

[B96] Nosofsky R. Zaki S. (2002). Exemplar and prototype models revisited: response strategies, selective attention, and stimulus generalization. *J. Exp. Psychol. Learn. Mem. Cogn.* 28 924–940 12219799

[B97] O’Connor K. N. Petkov C. I. Sutter M. L. (2005). Adaptive stimulus optimization for auditory cortical neurons. *J. Neurophysiol.* 94 4051–4067 16135553 10.1152/jn.00046.2005

[B98] Ohki K. Chung S. Ch’ng Y. H. Kara P. Reid R. C. (2005). Functional imaging with cellular resolution reveals precise micro-architecture in visual cortex. *Nature* 433 597–603 15660108 10.1038/nature03274

[B99] Olshausen B. A. Field D. J. (2005). How close are we to understanding v1? *Neural Comput.* 17 1665–1699 15969914 10.1162/0899766054026639

[B100] Opfer J. Siegler R. (2007). Representational change and children's numerical estimation. *Cogn. Psychol.* 55 169–195 17462620 10.1016/j.cogpsych.2006.09.002

[B101] Optican L. M. Richmond B. J. (1987). Temporal encoding of two-dimensional patterns by single units in primate inferior temporal cortex. III. Information theoretic analysis. *J. Neurophysiol.* 57 147–161 10.1152/jn.1987.57.1.162 3559670

[B102] Oram M. W. Xiao D. Dritschel B. Payne K. R. (2002). The temporal resolution of neural codes: does response latency have a unique role? *Philos. Trans. R. Soc. Lond. B Biol. Sci.* 357 987–1001 12217170 10.1098/rstb.2002.1113 PMC1693013

[B103] Paninski L. (2004). Maximum likelihood estimation of cascade point-process neural encoding models. *Network* 15 243–262 15600233

[B104] Paninski L. (2005). Asymptotic theory of information-theoretic experimental design. *Neural Comput.* 17 1480–1507 15901405 10.1162/0899766053723032

[B105] Paninski L. Pillow J. Lewi J. (2007). Statistical models for neural encoding, decoding, and optimal stimulus design. *Prog. Brain Res.* 165 493–507 17925266 10.1016/S0079-6123(06)65031-0

[B106] Penny W. (2012). Comparing dynamic causal models using AIC, BIC and free energy. *Neuroimage* 59 319–330 21864690 10.1016/j.neuroimage.2011.07.039 PMC3200437

[B107] Pillow J. W. Shlens J. Paninski L. Sher A. Litke A. M. Chichilnisky E. J. (2008). Spatio-temporal correlations and visual signalling in a complete neuronal population. *Nature* 454 995–999 18650810 10.1038/nature07140 PMC2684455

[B108] Poggio T. Girosi F. (1990). Networks for approximation and learning. *Proc. IEEE* 78 1481–1497

[B109] Prenger R. Wu M. C.-K. David S. V. Gallant J. L. (2004). Nonlinear v1 responses to natural scenes revealed by neural network analysis. *Neural Netw.* 17 663–679 15288891 10.1016/j.neunet.2004.03.008

[B110] Prinz A. Abbott L. Marder E. (2004). The dynamic clamp comes of age. *Trends Neurosci.* 27 218–224 15046881 10.1016/j.tins.2004.02.004

[B111] Rabinowitz N. Willmore B. Schnupp J. King A. (2012). Spectrotemporal contrast kernels for neurons in primary auditory cortex. *J. Neurosci.* 32 11271–11284 22895711 10.1523/JNEUROSCI.1715-12.2012 PMC3542625

[B112] Ray S. Hsiao S. S. Crone N. E. Franaszczuk P. J. Niebur E. (2008). Effect of stimulus intensity on the spike-local field potential relationship in the secondary somatosensory cortex. *J. Neurosci.* 28 7334–7343 18632937 10.1523/JNEUROSCI.1588-08.2008 PMC2597587

[B113] Reiss L. A. J. Young E. D. (2005). Spectral edge sensitivity in neural circuits of the dorsal cochlear nucleus. *J. Neurosci.* 25 3680–3691 15814799 10.1523/JNEUROSCI.4963-04.2005 PMC6725373

[B114] Rieke F. Warland D. de Ruyter van Steveninck R. Bialek W. (1997). *Spikes: Exploring the Neural Code*. Cambridge: MIT Press

[B115] Riesenhuber M. Poggio T. (1999). Hierarchical models of object recognition in cortex. *Nat. Neurosci.* 2 1019–1025 10526343 10.1038/14819

[B116] Riesenhuber M. Poggio T. (2000). Models of object recognition. *Nat. Neurosci.* 3(Suppl.) 1199–1204 11127838 10.1038/81479

[B117] Rubin D. Hinton S. Wenzel A. (1999). The precise time course of retention. *J. Exp. Psychol.* 25 1161–1176

[B118] Rubin D. Wenzel A. (1996). One hundred years of forgetting: a quantitative description of retention. *Psychol. Rev.* 103 734–760

[B119] Schinkel-Bielefeld N. David S. Shamma S. Butts D. (2012). Inferring the role of inhibition in auditory processing of complex natural stimuli. *J. Neurophysiol.* 107 3296–3307 22457454 10.1152/jn.01173.2011 PMC3378413

[B120] Schwarz G. (1978). Estimating the dimension of a model. *Ann. Stat.* 6 461–464

[B121] Seung H. Sompolinsky H. (1993). Simple models for reading neuronal population codes. *Proc. Natl. Acad. Sci. U.S.A.* 90 10749–10753 8248166 10.1073/pnas.90.22.10749 PMC47855

[B122] Shamma S. A. Fleshman J. W. Wiser P. R. Versnel H. (1993). Organization of response areas in ferret primary auditory cortex. *J. Neurophysiol.* 69 367–383 8459273 10.1152/jn.1993.69.2.367

[B123] Sharpee T. Miller K. Stryker M. (2008). On the importance of static nonlinearity in estimating spatiotemporal neural filters with natural stimuli. *J. Neurophysiol.* 99 2496–2509 18353910 10.1152/jn.01397.2007 PMC2877595

[B124] Sharpee T. Rust N. Bialek W. (2004). Analyzing neural responses to natural signals: maximally informative dimensions. *Neural Comput.* 16 223–250 15006095 10.1162/089976604322742010

[B125] Shepherd G. (2003). *The Synaptic Organization of the Brain*, 5 Edn. New York: Oxford University Press

[B126] Simoncelli E. (2003). Vision and the statistics of the visual environment. *Curr. Opin. Neurobiol.* 13 144–149 12744966 10.1016/s0959-4388(03)00047-3

[B127] Simoncelli E. P. Pillow J. W. Paninski L. Schwartz O. (2004). “Characterization of neural responses with stochastic stimuli,” in *The Cognitive Neurosciences III* ed. Gazzaniga M. (Cambridge, MA: MIT Press) 327–338

[B128] Snoeren P. R Puts M. J. H. (1997). Multiple parameter estimation in an adaptive psychometric method: MUEST, an extension of the quest method. *J. Math. Psychol.* 41 431–439 9473404 10.1006/jmps.1997.1188

[B129] Spall J. (2003). *Introduction to Stochastic Search and Optimization*. Hoboken, NJ: Wiley-Interscience

[B130] Steinmetz P. N. Roy A. Fitzgerald P. Hsiao S. Johnson K. Niebur E. (2000). Attention modulates synchronized neuronal firing in primate somatosensory cortex. *Nature* 404 187–190 10724171 10.1038/35004588

[B131] Stork D. G. Levinson J. Z. Albrecht D. F. DeValois R. L. Thorell L. G. (1982). Receptive fields and the optimal stimulus. *Science* 216 204–205 17736255 10.1126/science.216.4542.205

[B132] Sugiyama M. (2006). Active learning in approximately linear regression based on conditional expectation of generalization error. *J. Mach. Learn. Res.* 7 141–166

[B133] Tam W. (2012). *Adaptive Modeling of Marmoset Inferior Colliculus Neurons In Vivo*. PhD thesis, Department of Biomedical Engineering, Johns Hopkins University, Baltimore, MD

[B134] Tam W. Dekel E. DiMattina C. Young E. D. Zhang K. (2011). Using optimal experimental design for capturing parameters of neural networks in the inferior colliculus of the common marmoset. *Soc. Neurosci. Abstr.* 480 10

[B135] Tolhurst D. J. Movshon J. A. Dean A. F. (1983). The statistical reliability of signals in single neurons in cat and monkey visual cortex. *Vis. Res.* 23 775–85 6623937 10.1016/0042-6989(83)90200-6

[B136] Touryan J. Lau B. Dan Y. (2002). Isolation of relevant visual features from random stimuli for cortical complex cells. *J. Neurosci.* 22 10811–10818 12486174 10.1523/JNEUROSCI.22-24-10811.2002 PMC6758424

[B137] Tzanakou E. Michalak R. Harth E. (1979). The alopex process: visual receptive fields by response feedback. *Biol. Cybern.* 35 161–174 518937 10.1007/BF00337061

[B138] Ulanovsky N. Las L. Nelken I. (2003). Processing of low-probability sounds by cortical neurons. *Nat. Neurosci.* 6 391–398 12652303 10.1038/nn1032

[B139] VanRullen R. Guyonneau R. Thorpe S. J. (2005). Spike times make sense. *Trends Neurosci.* 28 1–4 15626490 10.1016/j.tins.2004.10.010

[B140] Victor J. D. Purpura K. P. (1996). Nature and precision of temporal coding in visual cortex: a metric-space analysis. *J. Neurophysiol.* 76 1310–1326 8871238 10.1152/jn.1996.76.2.1310

[B141] Vladusich T. Lucassen M. P. Cornelissen F. W. (2006). Do cortical neurons process luminance or contrast to encode surface properties? *J. Neurophysiol.* 95 2638–2649 16381807 10.1152/jn.01016.2005

[B142] Wang Z. Bovik A. (2006). *Modern Image Quality Assessment*. New York: Morgan & Claypool Publishers

[B143] Wang Z. Simoncelli E. P. (2008). Maximum differentiation (MAD) competition: a methodology for comparing computational models of perceptual quantities. *J. Vis.* 8 8.1–8.13 18831621 10.1167/8.12.8 PMC4143340

[B144] Watson A. B. Pelli D. G. (1983). Quest: a Bayesian adaptive psychometric method. *Percept. Psychophys.* 33 113–20 6844102 10.3758/bf03202828

[B145] Wei H. Amari S.-I. (2008). Dynamics of learning near singularities in radial basis function networks. *Neural Netw.* 21 989–1005 18693082 10.1016/j.neunet.2008.06.017

[B146] Wei H. Zhang J. Cousseau F. Ozeki T. Amari S.-I. (2008). Dynamics of learning near singularities in layered networks. *Neural Comput.* 20 813–843 18045020 10.1162/neco.2007.12-06-414

[B147] Willett R. Nowak R. (2007). Minimax optimal level-set estimation. *IEEE Trans. Image Proc.* 16 2965 10.1109/tip.2007.910175 18092596

[B148] Wixted J. Ebbesen E. (1991). On the form of forgetting. *Psychol. Sci.* 2 409

[B149] Wu M. C.-K. David S. V. Gallant J. L. (2006). Complete functional characterization of sensory neurons by system identification. *Annu. Rev. Neurosci.* 29 477–505 16776594 10.1146/annurev.neuro.29.051605.113024

[B150] Yamane Y. Carlson E. T. Bowman K. C. Wang Z. Connor C. E. (2008). A neural code for three-dimensional object shape in macaque inferotemporal cortex. *Nat. Neurosci.* 11 1352–60 18836443 10.1038/nn.2202 PMC2725445

[B151] Yarrow S. Challis E. Series P. (2012). Fisher and Shannon information in finite neural populations. *Neural Comput.* 24 1740–1780 22428594 10.1162/NECO_a_00292

[B152] Young E. D. Yu J. J Reiss L. A. J. (2005). Non-linearities and the representation of auditory spectra. *Int. Rev. Neurobiol.* 70 135–68 16472634 10.1016/S0074-7742(05)70005-2

[B153] Yu J. J. Young E. D. (2000). Linear and nonlinear pathways of spectral information transmission in the cochlear nucleus. *Proc. Natl. Acad. Sci. U.S.A.* 97 11780–11786 11050209 10.1073/pnas.97.22.11780 PMC34349

[B154] Zhang K. Sejnowski T. J. (1999). Neuronal tuning: to sharpen or broaden? *Neural Comput.* 11 75–84 9950722 10.1162/089976699300016809

